# Myostatin/Activin-A Signaling in the Vessel Wall and Vascular Calcification

**DOI:** 10.3390/cells10082070

**Published:** 2021-08-12

**Authors:** Pasquale Esposito, Daniela Verzola, Daniela Picciotto, Leda Cipriani, Francesca Viazzi, Giacomo Garibotto

**Affiliations:** 1 Department of Internal Medicine, University of Genova, 16132 Genova, Italy; pasquale.esposito@unige.it (P.E.); daverz@libero.it (D.V.); cipriani.leda@gmail.com (L.C.); francesca.viazzi@unige.it (F.V.); 2 IRCCS Ospedale Policlinico San Martino, Clinica Nefrologica, Dialisi, Trapianto, 16132 Genova, Italy; daniela.picciotto@hsanmartino.it

**Keywords:** myostatin, activin A, activin receptors, atherosclerosis, vascular calcification, CKD-MBD

## Abstract

A current hypothesis is that transforming growth factor-β signaling ligands, such as activin-A and myostatin, play a role in vascular damage in atherosclerosis and chronic kidney disease (CKD). Myostatin and activin-A bind with different affinity the activin receptors (type I or II), activating distinct intracellular signaling pathways and finally leading to modulation of gene expression. Myostatin and activin-A are expressed by different cell types and tissues, including muscle, kidney, reproductive system, immune cells, heart, and vessels, where they exert pleiotropic effects. In arterial vessels, experimental evidence indicates that myostatin may mostly promote vascular inflammation and premature aging, while activin-A is involved in the pathogenesis of vascular calcification and CKD-related mineral bone disorders. In this review, we discuss novel insights into the biology and physiology of the role played by myostatin and activin in the vascular wall, focusing on the experimental and clinical data, which suggest the involvement of these molecules in vascular remodeling and calcification processes. Moreover, we describe the strategies that have been used to modulate the activin downward signal. Understanding the role of myostatin/activin signaling in vascular disease and bone metabolism may provide novel therapeutic opportunities to improve the treatment of conditions still associated with high morbidity and mortality.

## 1. Introduction

Vascular disease, which may present with different pathological and clinical pictures, including arteriosclerosis, atherosclerosis, aortic aneurysms and vascular calcification, is a main cause of increased cardiovascular (CV) morbidity and mortality in aging and very common diseases, such as diabetes and chronic kidney disease (CKD) [1]. Experimental evidence shows a prominent contribution of vascular smooth muscle cell (VSMC) plasticity in vascular remodeling during aging [2]. Mechanisms and mediators leading to VSMC aging include mechano-stimuli, chronic inflammation, apoptosis, epigenetic events and calcification; these factors promote aging-induced changes in the VSMC functional pattern, including the ability to contract and assemble extracellular matrix proteins. This age-induced arterial remodeling identifies arteriosclerosis and provides a major risk for the development of atherosclerosis [3]. Despite recent advances in the understanding of the biology and pathogenesis of vascular damage in several conditions, including CKD, no satisfactory therapeutic strategies have been defined, yet [4,5].

This observation underlines the need to better clarify pathogenetic mechanisms of vascular disease to identify new potential therapeutic targets. In this view, much attention has been paid to the role of members of the transforming growth factor-β (TGF-β) superfamily [6].

Myostatin and activin-A are two of thirty-three members of the TGF-β family. This group of proteins, which, in addition to growth differentiation factors (GDFs) and TGF-βs, also includes also inhibins and bone morphogenetic proteins (BMPs), may regulate vascular calcification by influencing the plasticity of multipotent progenitor lineages associated with vessels [7].

Among the members of the TGF-β family, both myostatin (Mstn), a potent inhibitor of skeletal muscle development, and activin-A (Act-A), a multifunctional growth and differentiation factor, have been recently studied as potential regulators of vascular biology. However, available evidence suggests that these molecules show different activity profiles in arterial vessels. So, Mstn may have a prominent role in promoting vascular senescence and inflammation, whereas Act-A appears more implicated in the pathogenesis of vascular calcifications and mineral disorders [8].

In this review, we discuss the biology and physiology of Mstn and Act-A in the vascular wall, focusing on the experimental and clinical data describing the involvement of these TGF-β ligands in the processes of the maintenance of the vascular wall, the calcification processes and CKD-related mineral bone disorder (CKD-MBD). Moreover, we discuss recent experimental and clinical approaches implemented to modulate Act-A downward pathways, including the use of antagonists and receptor ligand traps.

## 2. Myostatin/Activin-A Signaling: Basic Biology and Functions

### 2.1. Mstn Biology

Mstn/GDF8 is synthesized as an inactive 375 KDa precursor (pre-pro Mstn), bearing at N-terminal domain a hydrophobic core of 24 amino acids (signal-peptide) and at the C-terminal region, 9 cysteine residues and a proteolytic processing site RSSR (Arg-Ser-Arg-Arg) recognized by furin, a calcium-dependent serine protease [9]. The proteolytic cleavage produces a 36/40 kDa latency-associated peptide (LAP) and a 12.5/26 kDa mature peptide, corresponding to a C-terminal monomer or dimer, respectively, that is the biologically active molecule. The non-covalent interaction with LAP retains Mstn in a latent state that cannot engage its receptor [10]. Only the subsequent proteolytic cleavage by BMP1/Tolloid family metalloproteases releases active Mstn [11].

### 2.2. Mstn Signaling and Functions

**Figure 1:**
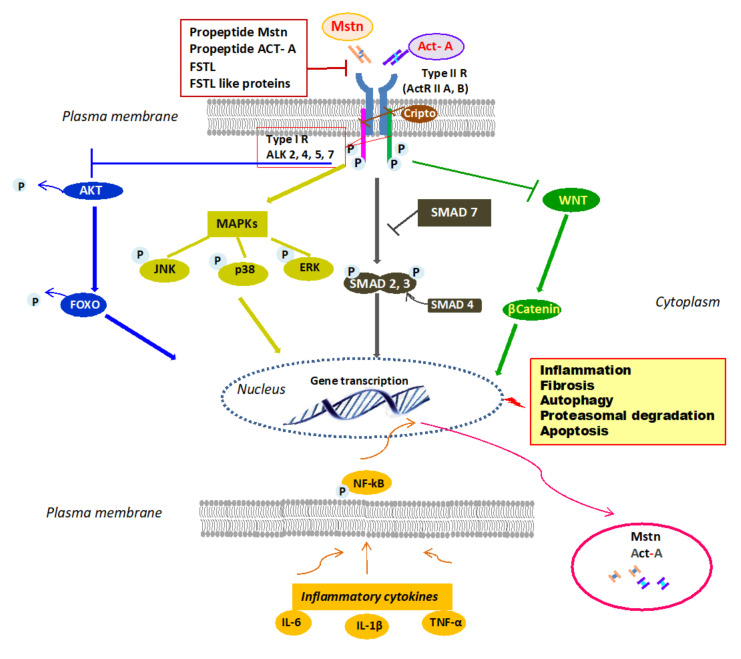
Mstn/Act-A cell signaling. Mstn/Act-A bind to ActRIIB/A on the plasma membrane, which leads to activation by phosphorylation of type-1 ALK 2, 4, 5, or 7. This in turn induces phosphorylation of SMAD2 and SMAD3 and the involvement of SMAD4 into a SMAD complex that translocates into the nucleus and elicits transcription of gene targets. SMAD7 inhibits SMAD pathway. Mstn and Act-A block AKT phosphorylation and consequently, dephosphorylated FOXO can enter the nucleus and promote the transcription and expression of atrophy specific genes. Mstn and Act-A can also signal through MAPK activation and WNT inhibition. The binding to the receptor is controlled by Mstn–Act-A propeptides and by circulating antagonists. Lastly, the ActRII/ActRIB receptor complex is inhibited by Cripto, a small GPI-anchored signaling protein. Inflammatory milieu contributes to Mstn and Act-A expression through NF-kB. Abbreviations: Act-A = activin A, ActRIIB/A = activin receptor type IIB, IIA; ALK = type-1 activin receptor serine kinase2, 4, 5, or 7; Mstn = myostatin; ERK = extracellular signal-regulated kinases; FOXO = forkhead box transcription factor; FSTL = follistatin; GPI = glycosylphosphatidylinositol-linked membrane-associated protein; IL-6 = interleukin-6; IL-1β = interleukin-1β; JNK = c-Jun N-terminal kinase; MAPKs = mitogen-activated protein kinases; NF-KB = nuclear factor kappa-light-chain-enhancer of activated B cells; P = phosphorylated; SMAD= small mothers against decapentaplegic; TNF-α = tumor necrosis factor-α; WNT = portmanteau of Wingless and integrated (WNT)/b-catenin pathway.

Mstn signals through a heteromeric complex of type I and type II receptors that are transmembrane proteins of approximately 55 and 70 KDa, respectively [12] (Figure 1), differing in level of sequence homology within the kinase domains and the presence of a highly conserved glycine–serine-rich (GS) domain in the cytoplasmic region of the type I receptor [13]. Mstn receptor engagement and its downward signaling are negatively regulated by the Mstn prodomain and by antagonists, including follistatin (FSTL) [14], follistatin-like 3 (FSTL3), GDF-associated serum protein-1 (GASP-1) [15] and Cripto, a small glycosylphosphatidylinositol-linked membrane-associated protein (GPI)-anchored signaling protein [16,17]. Firstly, Mstn binds to activin receptor IIB (ActRIIB) and then, this complex phosphorylates a type I activin receptor-like kinase-4 (ALK4) (myogenic cells), or -5 (ALK5) (nonmyogenic cells) [18]. This, in turn, phosphorylates small mothers against decapentaplegic (SMAD)2 and SMAD3 which recruit SMAD4, forming a SMAD2/3/4 complex that enters into the nucleus, regulating gene expression both positively and negatively [19]. SMAD7 works as a negative feedback inhibitor for the SMAD signal pathway [20]. Mainly, the canonical SMAD pathway mediates the effects of Mstn on myogenesis and muscle atrophy, upregulating the ubiquitin-proteasome system (UPS) synergistically with forkhead box transcription factors (FOXOs) and inhibiting the anabolic PI3-K/AKT/mTOR pathway [21]. However, Mstn can activate mitogen-activated protein kinases (MAPKs) that inhibit myoblast proliferation and differentiation [22] and promote an inflammatory milieu stimulating the expression of inflammatory cytokines [23]. On the other hand, inflammatory cytokines such as TNFα can induce Mstn expression through NF-kB [24]. Differently, Mstn has inhibitory effects on the Wnt/β-catenin pathway, thereby blunting satellite cell proliferation [25]. Mstn can exert pleiotropic regulatory effects. It has been found primarily expressed by animal and human skeletal muscle cells, where it limits muscle growth [26], inhibiting myogenesis and contributing to muscle loss through the activation of proteolysis and autophagy, mainly by upregulation of the UPS [27,28]. The negative effects of Mstn on muscle growth have been involved in the pathogenesis of age-related sarcopenia [29], as well as in cachexia associated with cancer [30], chronic kidney disease [31], and heart failure [32]. However, beyond regulating skeletal muscle growth, subsequent evidence has proved that Mstn is implicated in many physiological and pathological processes at the systemic level, including energy metabolism, the development of obesity, and insulin resistance [33,34]. Moreover, Mstn may play a role in the pathogenesis of diabetic nephropathy and heart failure [35]. In human proximal tubule cells, Mstn promotes proximal tubule activation and intracellular reactive oxygen species (ROS) release by upregulating NADPH oxidase. In patients with diabetic nephropathy, Mstn is associated with tubulointerstitial infiltrates and fibrotic areas [36]. The effects of Mstn on bone are less studied. Studies of Mstn-knockout mice revealed that early bone regeneration and inhibition of Mstn leads to an increase in osteogenesis. Mstn-/- mice showed an increase in density, strength, and bone mineralization [37]. In clinical studies, inhibition of Mstn increased the osteogenic potential and bone mineralization in patients with diabetes mellitus [38].

### 2.3. Act-A Biology

Act-A is a homodimer composed of 2 βA subunits linked with disulfide bonds. Act-A is produced as a larger precursor that is cleaved at amino-terminal sequence, releasing the mature 25 KDa carboxy-terminal bioactive ligand [39]. Differently from Mstn, which circulates as a latent precursor complex, the activin propeptide sequence has a weak affinity for the mature dimer, is easily displaced, and does not interfere with receptor binding. The subunits of Act-A are the products of the inhibin beta A gene, because of their original identification as subunits of the gonadal hormone, inhibin [40]. 

### 2.4. Act-A Signaling and Functions

Similarly to Mstn, Act-A signals by binding with high affinity to activin receptor type llA (ActRllA) or less so to activin receptor type IIB followed by the recruitment of the ActRI (ALK4, ALK7, or ALK2) [41] (Figure 1). Subsequently, the canonical SMAD pathway is activated. However, Act-A can induce MAPKs, which affect cell migration and differentiation [2,8], and controls the Wnt signaling pathway involved in developmental and injury processes [42]. Act-A action is regulated by several molecules. At the extracellular level, FSTL binds to Act-A with high affinity and prevents the engagement of type II receptors [43] and inhibin, binding to betaglycan, a type III TGF-B receptor, and then to ActRII, and competes with Act-A for the receptor site [44]. At the intracellular level, BAMBI (BMP and activin membrane-bound inhibitor), a transmembrane pseudoreceptor structurally similar to type I receptors, inhibits activin signaling [45] due to lacking the intracellular kinase domain. However, Cripto reduces Act-A efficacy, inhibiting the ability of the activin/ActRII complex to recruit the type I receptor, thereby inhibiting the activin downstream signaling pathway [17]. Act-A was first described as gonadal protein stimulating FSH secretion by the pituitary gland [46]. Then, it is expressed in the embryonal ovary, uterus, and testis, and in glands such as the breast and prostate. Interestingly, Act-A is also present in the human placenta, amnion, and chorion, and its levels are elevated in pregnant women [47]. However, apart from exerting diverse biological functions in the reproductive tract, Act-A and its receptors have been fully characterized in virtually all body systems [48]. Act-A is strongly expressed in different compartments of the central nervous system, where it seems to exert neuroprotective effects [49]. In addition, Act-A is present in both developing and adult heart, kidney, lung, and gastrointestinal tract [50]. In the heart, Act-A (and ActRs) may influence cardiomyocyte differentiation and remodeling after different kinds of injuries [51]. In the kidney, Act-A exerts a profibrotic effect, both during organ development and following acute and chronic damage [52,53]. Moreover, Act-A operates in concert with Mstn in negatively regulating muscle growth and may play a significant role in bone remodeling [54]. In bone, Act-A is secreted by osteoblasts and during bone matrix resorption by osteoclasts [55]. Different animal models showed that Act-A induces osteoblastogenesis, osteoclastogenesis, chondrogenesis, and collagen synthesis [56]. Coherently, inhibition of Act-A signaling obtained by administration of soluble ActRIIA, or the use of a ligand trap, was effective in preventing muscle wasting in different mouse models of experimental CKD and promoted osteogenesis and increased bone mass in healthy mice and primates [57,58]. Finally, Act-A modulates innate and adaptive immune mechanisms and mediates inflammatory responses [59]. Most immune cell types, including macrophages and T and B lymphocytes, can produce and respond to Act-A, and in inflammatory conditions, high levels of interleukin (IL)-1β and tumor necrosis factor-alpha (TNF-α) can promote, through NF-kB, Act-A expression and secretion, boosting the inflammatory process [60].

Furthermore, Act-A is proapoptotic in several cells, such as hepatocytes [61], renal proximal tubular cells [62], B cells [63], chronic myeloid leukaemia cells [64], and cardiac myocytes [65].

## 3. Myostatin and Activin-A in the Vessel Wall

Interestingly, while both Mstn and Act-A have been found expressed in the vascular wall, it seems that they present a different spectrum of effects on vessels. In particular, available evidence, below discussed in detail, suggests that while Mstn and Act-A may have opposite effects on promoting atherosclerosis, Act-A appears more implicated in the pathogenesis of vascular calcification [8]. 

### 3.1. Myostatin: A Role in Arterial Remodeling, VSMC Proliferation, and Accelerated Aging in Atherosclerotic Disease?

Atherosclerosis represents a dynamic and inflammatory process that takes place in large and medium-sized elastic and muscular arteries; it is the result of a complex interplay of risk factors and vascular insults that ultimately lead to intimal thickening and the creation of an early fibroatheroma that can progressively evolve into a thin cap fibroatheroma at high risk of rupture. Many actors are involved in atherogenesis, including VSMCs, endothelial cells (ECs), and monocytes. 

In particular, a key step is represented by the phenotypic shift of VSMCs that lose their contractile function and begin to proliferate, migrate from the media to the intima, and release chemokines and cytokines that in turn contribute to perpetuate the inflammatory proatherogenic vascular milieu [66].

There is accumulating evidence that Mstn plays a role in the pathophysiology of accelerated aging, atherosclerosis, and vascular remodeling. 

#### 3.1.1. Experimental Evidence

**Figure 2:**
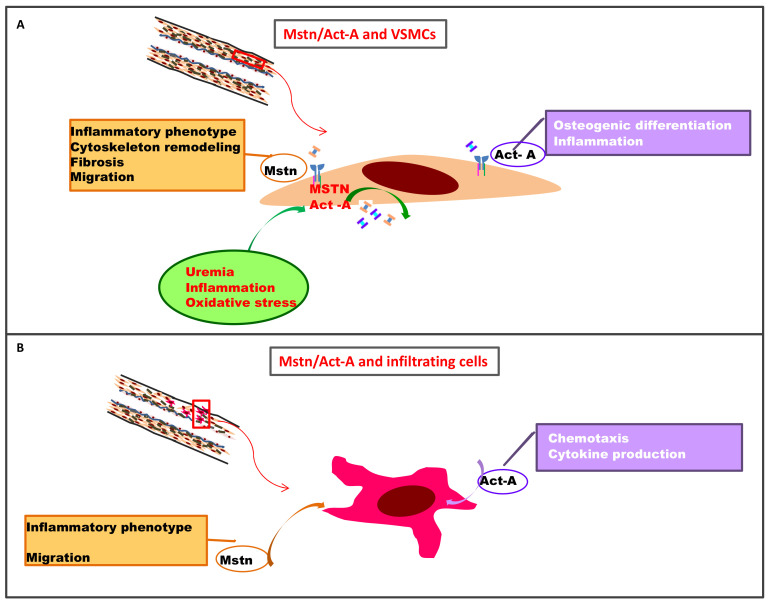
Role of Mstn and Act-A in vascular lesions: (**A**) In VSMCs, uremia, oxidative stress, and inflammation are some of the possible triggers of Mstn and Act-A expression, which could contribute to the atherosclerotic process. Mstn has a salient role in VSMC phenotypic modification, inducing cytoskeleton remodeling, migration, and inflammatory and pro-fibrotic molecule expression. On the other hand, Act-A is more involved with VSMC transformation into osteoblast-like cells responsible for medial calcification; (**B**) In atherosclerotic lesions, Mstn and Act-A infiltrating positive cells (monocytes, macrophages) are present. At that level, Mstn and Act-A may modulate inflammatory response and cell migratory capability. Abbreviations: Act-A = activin-A; Mstn = myostatin; VSMCs = vascular smooth muscle cells.

Myostatin interacts with many different types of vascular cells, inducing phenotypic modifications contributing to the atherosclerotic process (Figure 2). 

The exposure of aortic endothelial cells to Mstn activates TGF-β signaling, decreases endothelial NO synthase (eNOS) phosphorylation, and increases the expression of pro-atherogenic adhesion molecules ICAM-1 and VCAM-1 [67]. Moreover, in cultured rat VSMCs, Mstn decreases the proliferation rate and smoothelin expression, induces cytoskeletal rearrangement, increases the migratory rate, and upregulates chemokine expression [68]. In particular, VSMCs exposed to Mstn in vitro upregulate, via JNK activation, C-C chemokine receptor 2 (CCR2), and monocyte chemoattractant protein-1 (MCP1), which acts as a chemoattractant factor inducing the migration of monocytes into the vascular wall, thus accelerating atherogenesis. Notably, MCP1-treated monocytes express Mstn, suggesting a loop between MCP1 and Mstn [69]. Further proof of involvement of Mstn in the atherosclerotic process is provided by the observation that in low-density lipoprotein receptor-deficient mice, the genetic inactivation of Mstn can blunt the progression of diet-induced atherosclerosis [70]. 

Over the last few years, interest has grown in the research field regarding the short non-coding RNA molecules (microRNAs) as potential modulators of vascular lesions and remodeling processes. The inhibition of the 14q32microRNA cluster was found to be associated with reduced intimal hyperplasia and monocyte vascular accumulation in an experimental model of restenosis [71]. More recently, the same group observed an in vitro Mstn-mediated downregulation of 14q32 microRNAs miR-433-3p, miR-494-3p, and miR-495-3p in VSMCs, which leads to impaired cell proliferation but a preserved migration capacity. Furthermore, in a murine postinterventional restenosis model, myostatin inhibited the expression of both the 14q32microRNA cluster and the proliferation marker PCNA dose-specifically. However, Mstn infusion did not prevent restenosis, neointimal area, or lumen stenosis, and there was no effect on macrophage infiltration and activation. These findings suggest that both local inflammation and VSMC proliferation cooperate and are responsible for vascular remodeling and ultimately restenosis [72]. Mstn may play a broader role in vascular pathology that goes beyond atherosclerosis. For example, there is evidence of an Mstn-mediated impairment of vasomotor control. Indeed, the deletion of Mstn in obese mice leads to a restored endothelium mediated vasodilation [73], and the same effect, along with an improved cardiac ejection fraction, was observed in Mstn KO mice [74]. Another aspect of the Mstn effects on the vascular wall is its involvement in vascular senescence. Arterial wall aging is the result of a state of chronic inflammation associated with structural modifications and remodeling that lead to arterial stiffness and an augmented risk of atherosclerosis. In this process, similarly to atherogenesis, as reported above, Mstn is responsible for the phenotypic change of the VSMCs and the upregulation of the MCP1/CCR2 axis. Moreover, Mstn increases TGF-β levels and activates SMAD 2/3 pathways leading to increased collagen I production [2].

#### 3.1.2. Human Specimens

Consistent with experimental findings, myostatin expression is significantly increased in atherosclerotic vessels. Mstn expression was studied by our group in human aorta specimens representative of the full spectrum of atherosclerotic stages; in early stages of vascular remodeling, Mstn was observed in resident medial VSMCs, while in later stages of vascular lesions, Mstn overexpression was observed in the neointima, in neovessels and the media along the border, and within the plaques. In addition, infiltrating cells at the sites of atherosclerotic lesions and in the proximity of neovasa were strongly Mstn positive [68]. 

Elevated Mstn gene expression has also been observed in the vascular wall of CKD patients, where it is accompanied by the upregulation of atrogin-1 and MuRF-1, members of E3 ligases and major effectors of protein degradation, increased expression of CCL2/MCP-1, and decreased expression of cytoskeleton proteins and Klotho [75]. The Mstn pathway in CKD may also be activated by oxidative stress, inflammatory cytokines, and uremic toxins [76,77] that may act as inducers of Mstn and are involved in regulating phenotype modifications of VSMCs, closely linked to the occurrence of uremic vasculopathy [78]. These findings suggest that uremia increases Mstn gene expression and promotes vascular inflammation and aging in the vascular wall of patients with CKD. 

#### 3.1.3. Clinical Studies

Carotid-femoral pulse wave velocity (PWV) is the gold standard for the clinical assessment of arterial stiffness. PWV is an independent cardiovascular risk factor and predictor of future cardiovascular events [79]. In vivo evidence of the potential role of Mstn in the clinical setting comes from the recent MACISTE study that shows a direct and linear association between serum Mstn and the carotid-femoral PWV in healthy male adolescents. This finding suggests a premature pathogenetic role of Mstn in early vascular aging and arterial stiffness [80].

### 3.2. Activin A in the Vessel Wall

#### 3.2.1. Experimental Evidence

Act-A has been extensively studied as a potential modulator of the atherosclerotic process, following the observation that it influences the proliferation and differentiation of several cell types involved in atherogenesis, including ECs, macrophages, and VSMCs (Figure 2) [81,82].

In contrast to Mstn, data from in vitro studies showed that activin A may act as a negative regulator of the atherosclerotic process, determining the stabilization of atherosclerotic plaques. Indeed, activin inhibits EC proliferation and enhances the differentiation of monocytes into macrophages, blocking foam cell formation [83].

Interestingly, the effects of activin on the vascular structure were also studied in both human and experimental animal blood vessels. Engelse et al. used a human Act-A-expressing adenovirus (Ad-Act-A) to study the effect of activin A on neointima formation both in vitro using human saphenous vein organ cultures and in vivo in a murine model of cuffed femoral arteries [84].

In both experimental conditions (i.e., human vein and murine arteries) delivery of Act-A resulted in a significant inhibition of neointima formation. In the same experiment set, in cultured VSMCs of different origin, exposure to activin was associated with a significantly increased expression of smooth muscle (SM)–specific genes, such as SM22a and SM a-actin. So, it seems that, at the vascular level, Act-A promotes the differentiation of VSMCs toward a contractile, quiescent phenotype. 

Further experimental studies confirmed these findings and the potential to use Act-A expression to prevent vascular alterations in different conditions [85]. 

Interestingly, the effects of Act-A on cells involved in vascular wall biology and, in particular on ECs, seem also implicated in the pathogenesis of pulmonary arterial hypertension (PAH). Recently, Ryanto GRT et al. found overexpression of inhibin-β-a (INHBA), which encodes Act-A, by pulmonary endothelium in PAH [86]. In vitro, they also found that Act-A negatively regulates tube formation and EC functions by inducing bone morphogenetic protein receptor 2 internalization and degradation. Then, in experimental models, they observed that in the condition of hypoxia, INHBA overexpression, driven by VE-cadherin promoter (VEcad-INHBA-Tg), resulted in exacerbated pulmonary hypertension, accompanied by deteriorated right ventricular hypertrophy. 

Finally, they generated a mouse model selectively deficient in INHBA in endothelial cells (INHBA-ECKO mice). When compared with wild-type animals, INHBA-ECKO mice were protected against pulmonary vascular remodeling induced by hypoxia. Together, these data support the critical role of INHBA/Act-A on ECs and PAH progression, providing the understanding to design therapeutic strategies.

#### 3.2.2. Human Specimens

Studies on human tissue confirmed the expression of Act-A receptors in VSMCs, ECs, and macrophages, as well as the presence of Act-A in atherosclerotic lesions. In 1999, Engelse et al. studied the gene expression of Act-A, its physiological inhibitor FSTL, and activin receptors in human vascular tissue specimens that represented various stages of atherogenesis [87]. 

By in situ hybridization experiments, they revealed Act-A gene expression in endothelial cells, macrophages, and neointimal VSMCs from the early onset of atherogenesis. These results were also confirmed when they looked at the bioactive form of Act-A that was present in the neointimal region of the atherosclerotic vessel wall. Interestingly, in vitro studies on VSMC originated from the iliac artery and aorta of organ donors showed that Act-A induces the contractile, nonproliferative phenotype in cultured smooth muscle cells (characterized by an increased expression of SM a-actin and SM22a). Hence, these data reinforce the idea that Act-A is involved in plaque stabilization.

#### 3.2.3. Clinical Studies

Many clinical studies have investigated the relationship of Act-A serum levels with different features of vascular disease. Smith et al. evaluated Act-A serum levels and gene expression in peripheral blood mononuclear cells (PBMCs) in healthy subjects and patients with stable and unstable angina [88]. They found significantly increased serum levels of Act-A and PBMC expression in patients with cardiovascular disease (without any differences between stable and unstable angina), compared with healthy controls. Interestingly, in vitro exposure of PBMC to Act-A was associated with a dose-dependent decrease in the release of the inflammatory cytokines. These findings suggest an anti-inflammatory potential of Act-A in angina patients. Further researchers analyzed the relationship of Act-A circulating levels with infarct size and cardiac outcomes in patients with ischemic heart disease, showing that Act-A may have a prognostic and predictive significance [89,90]. Although interesting, these data remain to be confirmed in large and prospective studies. 

## 4. Myostatin and Activin-A in Vascular Calcification

The pathogenesis of vascular calcification, defined as the pathological deposition of mineral in the vascular system, does not encompass only a passive mineral step characterized by calcium phosphate deposition, but also an active and regulated cell-mediated process wherein cells in the arterial wall transdifferentiate to actively calcifying cells, resulting in a process resembling bone formation [91,92]. In addition, vascular calcification is considered a hallmark of vascular aging; consistently, it has been found that in apparently healthy adults, the prevalence of coronary calcification increases with age [4]. From a pathomorphological perspective, at least two types of calcifications can be distinguished based on location, association with plaque, and modality of formation [93]:

- calcification in arterial intimal layers in association with macrophages, lipids, and VSMCs, as in classical atherosclerosis;
- calcification in arterial medial layers, as a result of elastin fiber mineralization, VSMC degeneration, and upregulation of osteogenic programs as in CKD or diabetes, e.g., Möckenberg’s medial sclerosis of large arteries. There are some similarities, but also marked differences between intimal atherosclerotic and medial calcification; although inflammation and cytokine production are common to both types, osteogenic differentiation with metaplastic bone formation is only rarely implicated in intimal calcification, whereas it is often seen in medial calcification of peripheral arteries [94]. Hence, it is reasonable to hypothesize that the different types of vascular calcifications present distinct pathogenetic mechanisms. Experimental studies support this idea [95]; so, in a rabbit model, the induction of CKD by subtotal nephrectomy caused media degeneration with calcification in the absence of intimal calcification and lipid deposition [96]. Notably, in CKD, vascular calcification recapitulates many aspects of bone formation, and both processes share several pathogenic mechanisms. Furthermore, vascular calcification is frequently associated with MBD, and its progression is associated with decreased bone mineral density and altered bone turnover.

The calcification process is regulated by various inhibitors and promoters, including BMP/GDF ligands, members of the TGF-β superfamily. As members of the TGF-β superfamily exerting pleiotropic regulatory activities, Mstn/Act-A signaling also might potentially be involved in the vascular calcification process.

### 4.1. Myostatin and Vascular Calcification

Although Mstn has been found to promote vascular inflammation and VSMC cytoskeleton derangement, the direct relationship between Mstn and vascular calcification has been poorly investigated, and available data give inconclusive results. Thus, in the arterial wall of CKD patients, we did not find a correlation between tissue myostatin and calcium deposition as evaluated by immunohistochemistry [75]. In addition, in a cross-sectional study in a cohort of 71 hemodialysis (HD) patients, Lee et al. observed an inverse relationship between serum Mstn levels and higher degrees of aortic calcification observed in lumbar spine Rx [97]. Similar results were reported in the STRAMBO study, which investigated in a large cohort of healthy men the association between serum levels of Mstn and abdominal aortic calcifications evaluated through spine dual-energy X-ray absorptiometry. In this case, the authors confirmed that, at least in older men, total Mstn serum levels were inversely correlated with vascular calcifications [98].

### 4.2. Activin-A Signaling and Vascular Calcification

Recent observations strongly suggest that Act-A signaling plays a major role in the pathogenesis of vascular calcification and CKD-MBD progression [99].

#### 4.2.1. Experimental Studies

Preclinical studies have shown an upregulation of Act-A signaling in the kidney, bone, arterial vessels, and heart in CKD [100]. In a landmark study, Agapova et al. examined the role of ActRIIA signaling in CKD-related vascular disease, using a well-established high-fat-fed, diabetic, low-density lipoprotein receptor knockout (ldlr-/-) mouse model of CKD. In this model, both Act-A and ActRIIA protein levels and phosphorylated SMAD 2/3 levels were reduced in the aortas of mice with atherosclerotic calcification. The treatment with a ligand trap for ActRIIA, RAP-011 (a soluble extracellular domain of ActRIIA fused to a murine IgG-Fc fragment) resulted in increased aortic ActRIIA signaling, reduced expression of osteoblastic proteins, increased levels of aortic SM-22, and most notably, a significant reduction of aortic calcium deposition [101]. In the kidney, treatment with RAP-011 resulted in reduced expression of tissue Act-A and decreased p-SMAD2/3, which were associated with increased renal α-Klotho expression and decreased renal fibrosis and proteinuria. The potential therapeutic effects of modulation of ActRIIA signaling have been further studied by Williams et al., who tested RAP-011 treatment in a non-diabetic mice model of Alport syndrome, characterized by progressive CKD and CKD-MBD, presenting with renal osteodystrophy, vascular calcification, cardiac hypertrophy, and hyperparathyroidism. They found that RAP-011 did not directly affect ActRIIA expression, but significantly reduced vascular Act-A and p-SMAD2/3 levels, being associated with improvement of osteodystrophy and prevention of vascular calcifications. Moreover, RAP-011 attenuated renal interstitial fibrosis [100]. Overall looking at these results, it seems that regardless of the experimental model, which may present distinct levels of ActRIIA activation, the modulation of Act-A signaling could be effective in treating both vascular calcification and renal fibrosis. Considering the strict correlation among CKD, vascular calcification, and bone disorders, the potential for a therapeutic approach based on Act-A inhibition also comes from studies that explored the role of Act-A as a bone marker and as a modulator of bone metabolism in CKD [102]. Hence, in CKD models, systemic activation of ActRllA and Act-A levels exerted a positive SMAD-mediated regulatory effect on receptor activator of nuclear factor kappa-B ligand (RANKL), leading to osteoclastogenesis. This condition was associated with high-turnover osteopenia and increased bone resorption [103]. Furthermore inhibition of Act-A signaling with RAP-011 has been tested in normal and ovariectomized mice with established bone loss. In these models, treatment with RAP-011 increased bone formation, bone mass, and bone strength. Given these results, more recently, the effect of treatment with RAP-011 on bone metabolism has also been tested in CKD-MBD mouse models [104]. 

Sugatani et al., using the *ldlr-/-*high-fat-fed diabetic mouse model, found that RAP-011 treatment eliminated the CKD-induced increase in osteoclast and osteoblast numbers and bone resorption. Moreover, RAP-011 significantly increased bone volume, cortical bone area, and thickness. Interestingly, these effects were PTH-independent, suggesting that Act-A may exert peculiar regulatory effects on bone metabolism and confirming the possibility to consider modulation of Act-A signaling as a new therapeutic strategy in the treatment of CKD-MBD [105].

Moreover, an additional effect of Act-A signaling that could be linked to CKD-MBD and have a significant clinical impact is the role of this pathway in the regulation of erythropoiesis [106]. Indeed, in mouse models, RAP-011 treatment induced a significant increase in haematocrit, Hb, and RBC count. These effects were accompanied by a rapid stimulation of late-stage erythroid precursors in the bone marrow [107]. 

Finally, to broaden the discussion to the therapeutic approaches based on Act-A signaling modulation, it should be mentioned that administration of the recently developed ActRIIB:ALK4-Fc, a heterodimeric ligand-trapping fusion protein of the extracellular domains of ALK4 and ActRIIB, improved muscle dysfunction in murine models of neuromuscular disorders [108].

#### 4.2.2. Human Specimens and Clinical Studies

In humans, it has been found that Act-A produced by the kidney progressively increases in the blood of CKD patients [109]. Its serum levels increase early during CKD, before other bone markers, and correlate with bone turnover similar to intact PTH and FGF-23 [110]. Moreover, looking specifically at vascular calcification, in observational studies carotid intima-media thickness (cIMT), considered a clinical expression of the vascular calcification process, significantly correlated with Act-A serum levels, both in diabetes and CKD patients [111,112].

Finally, some clinical trials with pharmacological agents targeting Act-A signaling have been performed and are ongoing. Their results will be discussed in the next paragraphs. 

## 5. Activin-A Signaling Vascular and Soft Tissue Calcifications: Lessons from Fibrodysplasia Ossificans Progressiva (FOP)

A peculiar condition that could be paradigmatic to recognize the role of Act-A signaling in promoting ectopic calcification is FOP, a rare genetic disorder due to the mutation of the activin receptor 1/activin receptor-like kinase 2 (ACVR1/ALK2, c.617 G>A, R206H) gene encoding for BMP type-1 receptor (BMPR1) [113].

The hyperactivity and widespread dysregulation of the downstream BMP signaling pathway, mainly due to the receptor alteration, causes progressive heterotopic ossification of soft connective tissues, associated with endothelial–mesenchymal transitions. Interestingly, as one of the main pathogenic mechanisms responsible for FOP, the mutation confers to ACVR1 the ability to bind and transduce the signal mediated by Act-A. So, the binding of Act-A to the mutant receptor activates the downstream signaling through canonical SMAD 1/5/8 mediators [114]. To investigate the implication of these findings, Hatsell et al. developed a knock-in mouse model for FOP (Acvr1[R206H]FlEx). In this mouse model, Act-A injection triggered ectopic ossification, which was completely prevented by treatment with anti-Act-A antibodies [115]. These observations have provided the basis to consider Act-A modulation as a potential therapeutic strategy for FOP [116].

## 6. Clinical Therapeutic Developments 

Modulation of Mstn/Act-A signaling has been mainly used in experimental models to investigate the pathophysiology and functions of these pathways. 

Unfortunately, the pleiotropic actions of TGF-β superfamily components and the context-dependent effects of the signaling activated by the members of the superfamily are major challenges in therapeutical translation.

Due to their regulatory properties, these molecules have also been studied as potential therapeutic targets in different clinical conditions in humans. Considering the negative effects of Mstn on muscle growth, clinical studies have been performed mainly to evaluate whether the block of Mstn signaling might improve muscle mass and performance status in patients with sarcopenia and muscular dystrophy [117], whereas no data are available on the vascular effects of Mstn inhibition. It is interesting that one of these studies, a phase 2 trial of ACE-031 in boys with Duchenne’s muscle dystrophy, was prematurely terminated [118] following the occurrence of signs observed in patients with hereditary hemorrhagic telangiectasia (HHT), a disease caused by mutations in ALK-1, which blunts TGFβ receptor signaling in endothelial cells [119]. In addition, a more recently developed ligand trap, ActRIIA-Fc, blunts myostatin, activin, and GDF11 actions in endothelial cells, as well as arteriolar remodeling, while promoting vessel apoptosis in animal models of PAH [120]. These results suggest that Mstn, Act-A, and GDF-11 participate in blood vessel remodeling.

Moreover, based on the findings of preclinical studies, inhibition of Act-A signaling has been tested in the treatment of anemia. Luspatercept (ACE-536), an ActRIIB, with the modified extracellular domain of ActRIIB fused to the Fc fragment, after a promising phase 2 trial [121], was administered to myelodysplastic syndromes in a large phase 3 trial [122]. In these patients, luspatercept showed a sufficient safety profile, reducing the severity of anaemia and transfusion requirements. Similar results have been reported for sotatercept (ACE-011), the administration of which improved anemia in patients with both myelodysplastic syndrome and ß -thalassemia [123].

Interestingly, sotatercept was also tested in HD patients in two phase II, multicenter, randomized trials, REN-001 and REN-002 [124]. In these studies, the safety, tolerability, and effects of different dosages of sotatercept (from 0.1 to 0.7 mg/kg) on hemoglobin concentration were evaluated; in addition, in REN-001, the effects of sotatercept on bone mineral density (BMD) and abdominal aortic vascular calcification were also explored. The results of both studies showed an acceptable safety profile and effectiveness in achieving target hemoglobin response (>10 g/dL) when compared to placebo. Moreover, sotatercept showed a dose-dependent trend toward improving BMD and slowing the progression of abdominal aortic vascular calcifications. 

These findings provided the first clinical proof of the reliability of Act-A signaling as an effective therapeutic strategy in treating CKD-MBD and vascular calcification.

The results of the clinical trials with luspatercept and sotatercept are summarized in Table 1.

## 7. Conclusions

Recent studies suggest that Mstn and Act-A, along with other members of the TGF-β family, are implicated in the mechanisms of vascular aging and vascular disease.

The definition of the roles of Mstn and Act-A in the pathogenesis of atherosclerosis, vascular calcification, and CKD-MBD may provide the rationale to design original therapeutic strategies that could be complementary to current treatments that are often not fully satisfactory.

In this view, strategies modulating Act-A signaling, mainly based on receptor ligand traps, have been successfully tested in animal models, where they have shown effectiveness in treating muscle atrophy, vascular disease, renal fibrosis, high-turnover bone disease, and anemia. 

Some recent RCTs have proved the reliability of Act-A signaling as a new target for treating anemia (secondary to different diseases), bone loss, and vascular calcifications. Although these preliminary data are encouraging, additional preclinical investigations using alternative animal models and clinical studies targeting specific patient populations are needed to assess the safety and effectiveness of these new therapeutical approaches.

## Figures and Tables

**Table 1:** Clinical trials of sotatercept and luspatercept.

Name	Structure	Target	Indication	Phase Status [Ref]	Type	Patients n	Main Results
**Sotatercept** **(ACE-011** **ACVR2A-Fc)**	Extracellular domain ActRIIA + human IgG1 Fc domain	MSTN GDF11 ActivinsBMPs	Anemia	Phase 1 [125] Phase 2 [126] Phase 2 [124] Phase 2 [124]	RCT Open-label RCT Open-label	31 74 43 50	Dose-dependent increase in Hb levels, hematocrit, and RBC count Increased Hb levels in ESA-refractory MDS Target Hb levels achieved in higher proportion of treated patients than placebo group, with dose-dependent results
			Vascular calcification and bone mineral density disorders	Phase 1 [125] Phase 2 [124]	RCT RCT	31 43	Increased bone mineral density and biomarkers of bone formation Abdominal aortic vascular calcification slowed in a dose-related manner, less consistent data on BMD improvement
			Osteoporosis	Phase 2 [127]	RCT	48	Increased bone-specific ALP, decreased CTX
			Β-thalassemia	Phase 2 [123]	Open-label	46	Increased Hb levels, reduction of transfusion burden
			PAH	Phase 2 [128]	RCT	106	Reduction of pulmonary vascular resistance, improvement of 6-min walking test distance, lower pro-BNP levels
			Multiple myeloma	Phase 2 [129]	RCT	30	Anabolic improvement in BMD and bone formation
			Anemia CT-induced	Phase 2 [130]	RCT	55	Terminated early for slow recruitment
**Luspatercept** **(ACE-536** **ACVR2B-Fc)**	Extracellular domain ActRIIB + human IgG1 Fc domain	MSTN GDF11 Activins BMPs	Anemia, MDS	Phase 2 [121] Phase 3 [122]	Open-label RCT	58 229	Higher Hb levels, lower transfusion burden Trasfusion independence in 38% of the patients
			Β-thalassemia	Phase 2 [131] Phase 3 [132]	Open-label RCT	64 336	Reduction >20% in transfusion burden in 81% of cases Reduction >33% in transfusion burden in 70.5% of the patients, >50% in 40.2% of the patients

Abbreviations: ActRIIB/A = activin receptor type IIB/A; ALP = alkaline phosphatase; BMD = bone mineral density; BMP = bone morphogenetic proteins; CT = chemotherapy; CTX = carboxy-terminal collagen crosslinks; ESA = erythropoiesis-stimulating agents; GDF11= growth differentiation factor 11; Hb = hemoglobin; MSD = myelodysplastic syndrome; MSTN = Myostatin; PAH = pulmonary arterial hypertension; RBC = red blood cells; RCT = randomized controlled trial.
